# Supplementary material for: Demonstration of critical coupling in an active III-nitride microdisk photonic circuit on silicon
Source: Sci Rep. 2019 Dec 2;9:18095. doi: 10.1038/s41598-019-54416-3 (PMC6889465; doi:10.1038/s41598-019-54416-3)
Supplement: Supplementary file 1 — Supplementary Information [file 41598_2019_54416_MOESM1_ESM.pdf]

Supporting Information for Demonstration of critical coupling in an active III-nitride microdisk photonic circuit on silicon

Farsane Tabataba-Vakili[1], Laetitia Doyennette[3], Christelle Brimont[3], Thierry Guillet[3], Stéphanie Rennesson[4], Benjamin Damilano[4], Eric Frayssinet[4], Jean-Yves Duboz[4], Xavier Checoury[1], Sébastien Sauvage[1], Moustafa El Kurdi[1], Fabrice Semond[4], Bruno Gayral[2], and Philippe Boucaud[4,*]

[1]Centre de Nanosciences et de Nanotechnologies, CNRS, Univ. Paris-Sud, Université Paris-Saclay, F-91120 Palaiseau, France
[2]Univ. Grenoble Alpes, CEA, INAC-Pheliqs, 38000 Grenoble, France
[3]Laboratoire Charles Coulomb (L2C), Université de Montpellier, CNRS, Montpellier, France
[4]Université Côte d'Azur, CNRS, CRHEA, F-06560 Valbonne, France
[*]philippe.boucaud@crhea.cnrs.fr

ABSTRACT

Supporting information for Demonstration of critical coupling in an active III-nitride microdisk photonic circuit on silicon. Device characterization, FDTD simulations, maximum output power, and lasing and mode shift are discussed in more detail.

Device characterization

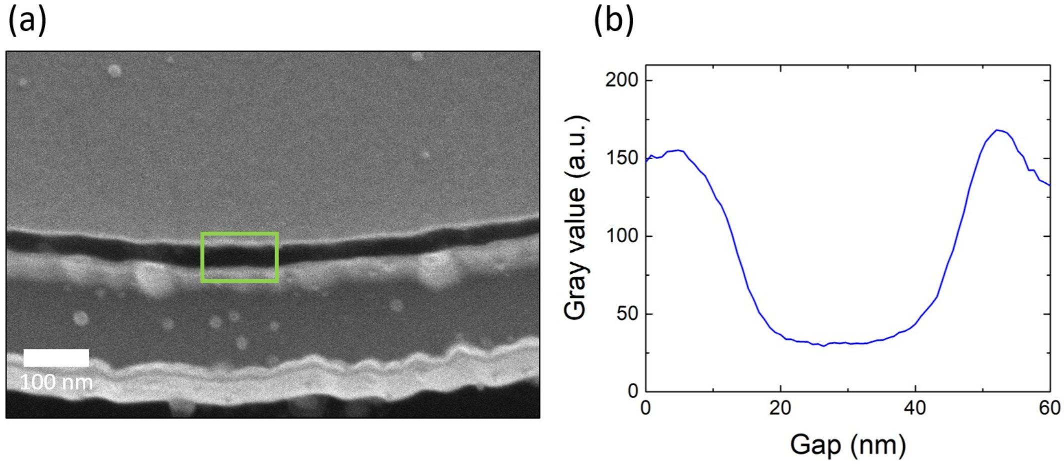

Figure S1. (a) SEM image of a nominally 50 nm gap and (b) gray scale profile along the vertical direction and integrated along the horizontal direction of the rectangle in (a), giving a gap of 45 nm at the top.

Figure S1 (a) shows an SEM image of a device with a gap of nominally 50 nm. The gray scale profile in Fig. S1 (b) is measured along the vertical direction and integrated along the horizontal direction of the rectangle in Fig. S1 (a). The measurement gives a gap of 45 nm at the top. With this method we determined the gaps provided in the main text.

FDTD simulations

Three-dimensional (3D) finite-difference time-domain (FDTD) simulations of devices with 5 μm diameter were performed with a Gaussian source placed in one end of the waveguide for transmission simulations. Fig. S2 shows the simulated transmitted radiative flux of a 5 μm diameter microdisk, $A = 90°$, $w = 170$ nm, and $g = 50$ nm. The emitted flux is measured at the end of

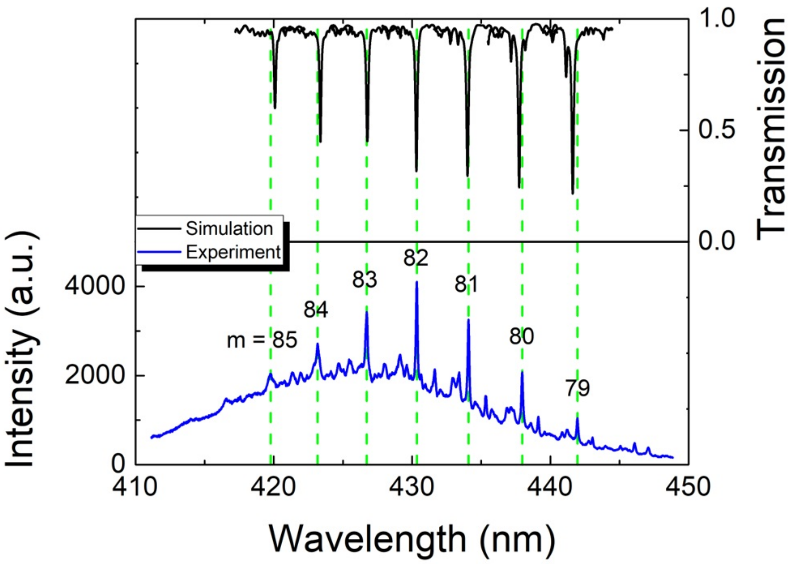

Figure S2. Simulated (top) and experimental (bottom) spectra of a device with $A = 90^\circ$ and $g = 50$ nm (simulated) and 45 nm (experimental). The azimuthal orders of the first-order radial modes are $m = 85$ to 79.

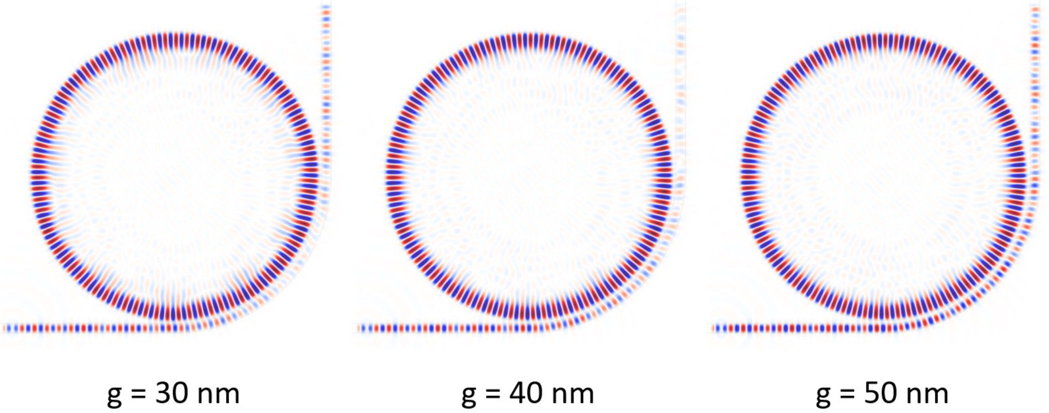

Figure S3. FDTD simulations of the H_z field for devices with 5 μm diameter, $w = 170$ nm, and $g = 30$ nm to 50 nm.

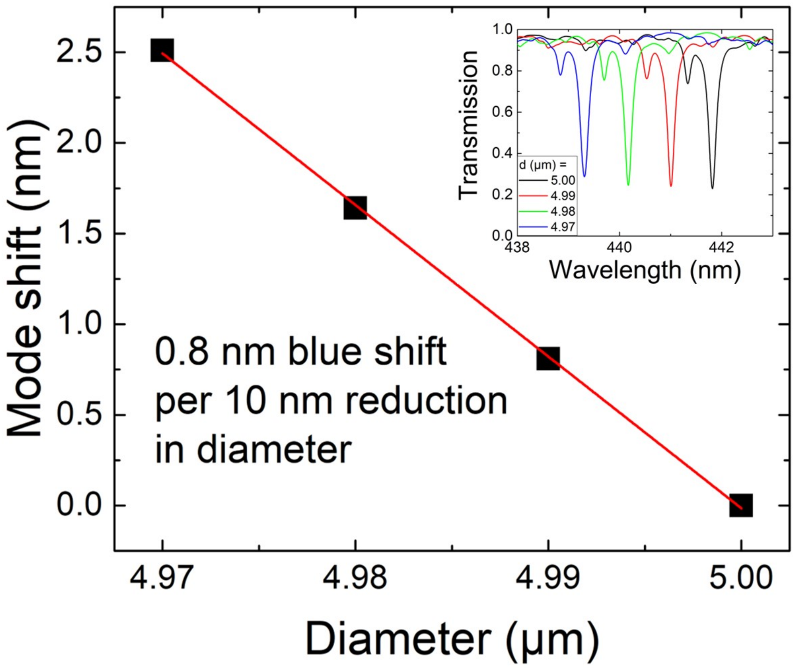

Figure S4. Mode shift over diameter. The linear fit gives a 0.8 nm blue shift per 10 nm reduction in diameter. The inset shows the FDTD transmission simulations of the 420 nm mode.

the waveguide and divided by the flux at the beginning of the waveguide to obtain the transmission. Refractive indices at 7 different wavelengths are used to take dispersion into account. The simulation is compared to the experimental CW spectrum (bottom) of Fig. 2(a) in the main text with $g = 45$ nm. The positions of first-order radial modes match well between simulation and experiment and the mode spacing is 3.6 to 4.0 nm. The theoretical mode spacing $\Delta\lambda$ is given by

$$\Delta\lambda = \frac{\lambda^2}{2\pi r n_g}, \tag{S1}$$

where n_g is the group index. For bulk AlN $n_g = 2.33$ and for bulk GaN $n_g = 3.23$ at $\lambda = 430$ nm. This gives an effective $n_{g,eff} = 3.2$ for the investigated microresonator and an expected mode spacing of $\Delta\lambda = 3.7$ nm, matching very well with the experimental and simulated results. The azimuthal orders of the modes are identified in Fig. S2 and are determined by counting the nodes in the H_z field. Modes of orders $m = 85$ to 79 are visible.

The simulated H_z field of devices with 5 μm diameter and $g = 30$ to 50 nm are depicted in Fig. S3. Good phase matching can be observed for $g = 40$ and 50 nm. The depicted mode is a first order radial mode with azimuthal order $m = 79$ at 441 nm.

The device geometry in the simulation is slightly different than for the fabricated devices. However, the region of interest, the coupling region is the same. The waveguide bends around the disk in a 90° angle at a distance g from the disk. The only thing missing in the simulation is the waveguide bending away from the disk in a region that is irrelevant for coupling, which will only provide some minor bending losses that we can neglect in our simulation as we are focusing on the coupling.

Figure S4 shows the mode shift over diameter, indicating that per 10 nm reduction in diameter the mode blue shifts by 0.8 nm. The inset shows the corresponding transmission simulations for a mode at 441 nm. This phenomenon is observed experimentally due to the proximity effect of the waveguide with decreasing gap during the fabrication process and is discussed in Fig. 6 in the main text.

Maximum output power

In order to determine the Q_C value where P_{out} becomes maximal for a given P_{pump} we calculate $\mathrm{d}P_{\text{out}}/\mathrm{d}Q_C = 0$, which gives

$$P_{\text{pump}} = F + \frac{BC}{Q_C^2} \cdot \frac{(Q_{\text{int}} + Q_C)^2}{C Q_{\text{int}}}. \tag{S2}$$

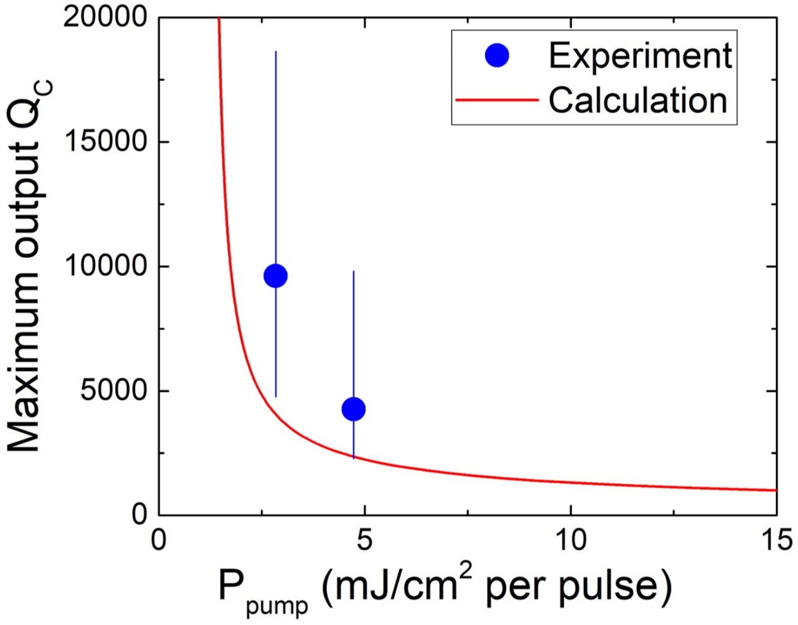

Figure S5. Maximum output Q_C as a function of P_{pump} according to equation (S2) and with the experimental values from Fig. 5 in the main text with error bars.

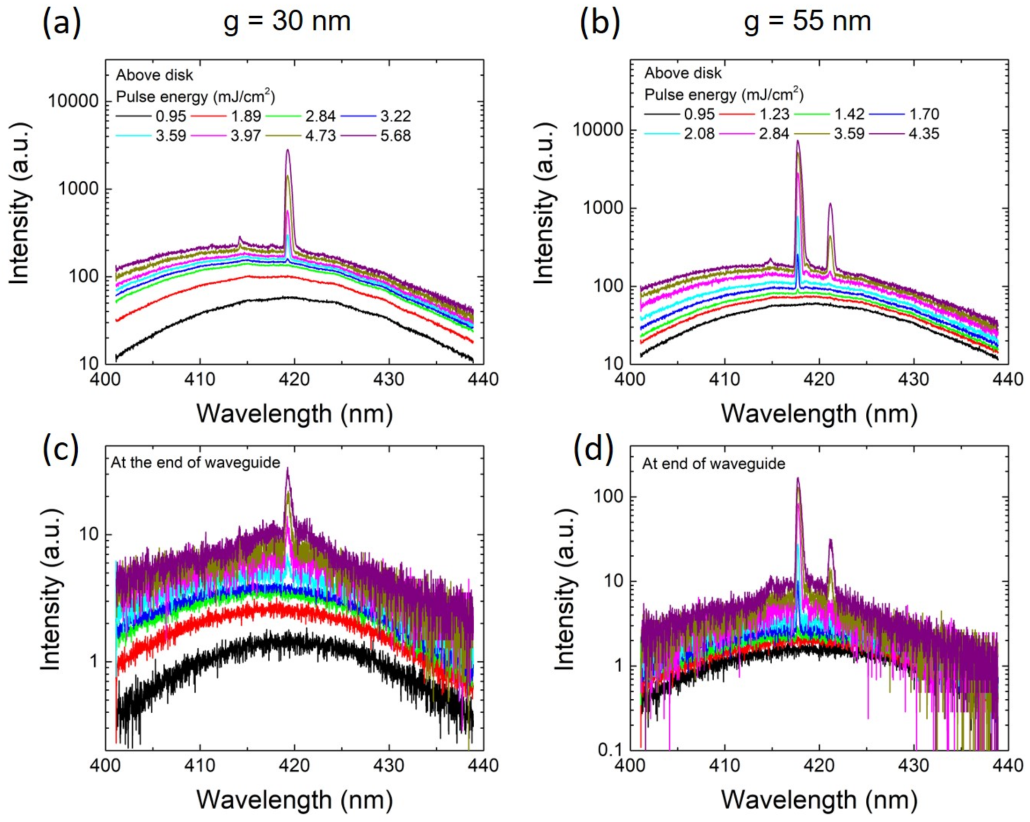

Figure S6. Lasing spectra measured above the disk for (a) $g = 30$ nm and (b) $g = 55$ nm and at the end of the waveguide for (c) $g = 30$ nm and (d) $g = 55$ nm.

Figure S5 shows the resulting maximum output Q_C over P_{pump}. In the experimentally investigated range of 2 to 5 mJ/cm^2 per pulse maximum P_{out} is achieved for Q_C values in the range of 7000 to 2000. The experimental values are taken from Fig. 5 in the main text. There are many parameters that go into the fit in Fig. S5, such as the fit of P_{th} over Q_{loaded} (Fig. 4 (d)), the fit of P_{out} over Q_C (Fig. 5), and Q_{int}, all of which have an uncertainty. The here shown fit is only intended to show a tendency based on the different parameters extracted from Fig. 4 and 5 and modelling.

Lasing and mode shift

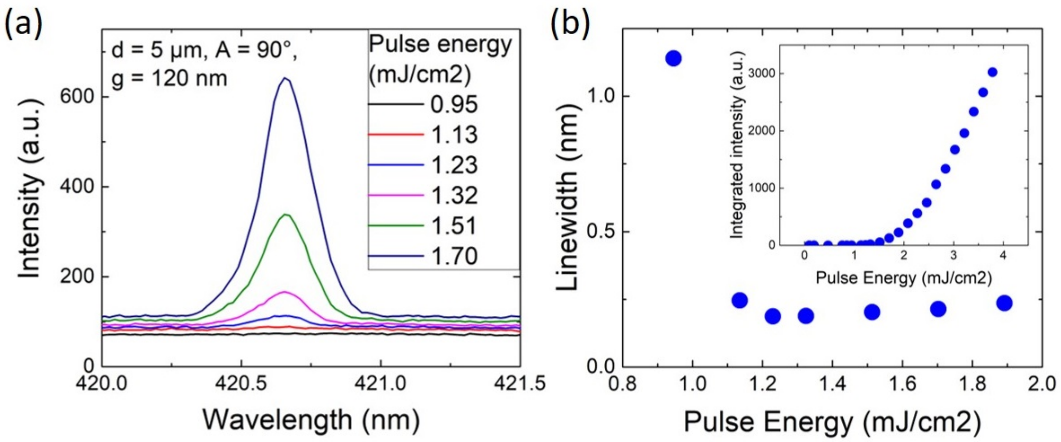

Figure S7. (a) Pulse-energy dependent spectra around threshold for a device with $A = 90°$ and $g = 120$ nm. The threshold is at 1.2 mJ/cm^2 per pulse. (b) Linewidth vs. pulse energy of the mode in (a) and (inset) integrated intensity vs. pulse energy for the same mode.

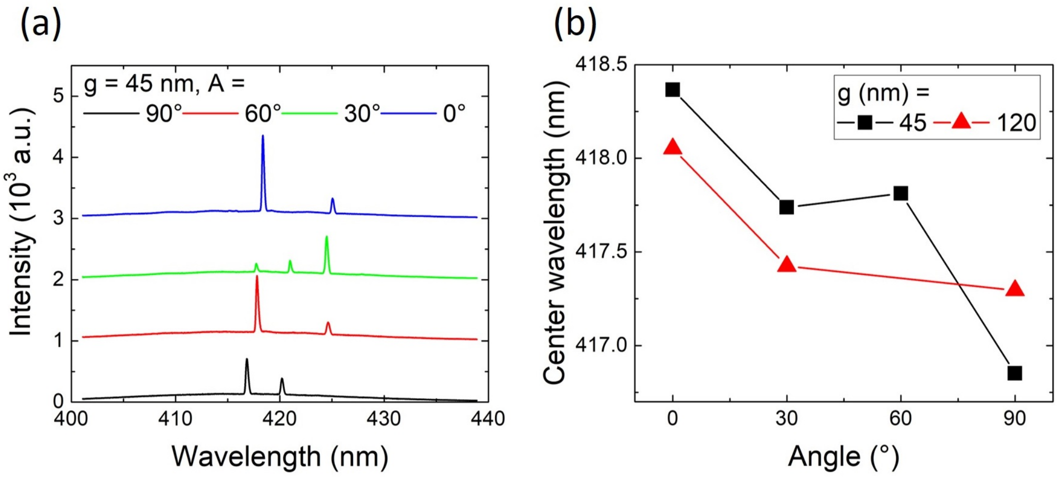

Figure S8. (a) Pulsed lasing spectra measured at the disk for devices with $g = 45$ nm for different angles. (b) Peak wavelength as a function of angle for $g = 45$ nm and $g = 120$ nm.

Pulse-energy density dependent measurements of devices with $g = 30$ nm and $g = 55$ nm are shown in Fig. S6 measured both above the disk and at the end of the waveguide. It can be clearly seen that the threshold decreases with increasing gap, as is discussed in the main text in respect to Fig. 4.

Figure S7 (a) shows pulse-energy dependent spectra around the threshold for a device with $A = 90°$ and $g = 120$ nm. The threshold is determined to be 1.2 mJ/cm^2 per pulse, which corresponds to 300 kW/cm^2. The linewidth over pulse energy is shown Fig. S7 (b), indicating a linewidth narrowing of more than a factor of 2. The inset in Fig. S7 (b) shows the integrated intensity over pulse energy.

Figure S8 (a) shows spectra for devices with $g = 45$ nm and angles from $A = 0°$ to $90°$. Figure S8 (b) shows the peak wavelength vs. A for $g = 45$ and 120 nm. For small angles a constant 0.3 nm blue shift is observed when going from $g = 45$ to

120 nm, due to the change in effective refractive index. For small angles the diameter reduction due to waveguide proximity is negligible. At $A = 90^\circ$ a 0.4 nm red shift is observed when going from $g = 45$ to 120 nm due to the increase in disk diameter of approximately 5 nm, given by Fig. S4. With increasing angle a blue shift is observed due to a reduction in disk diameter, which is more pronounced at a smaller gap size. The blue shifts of 2 nm at $g = 45$ nm and 1 nm at $g = 120$ nm correspond to 20 nm and 10 nm reduction in disk diameter, respectively.
